# Distinct genomic features between osteosarcomas firstly metastasing to bone and to lung

**DOI:** 10.1016/j.heliyon.2023.e15527

**Published:** 2023-04-17

**Authors:** Lu Xie, Zhenyu Cai, Hezhe Lu, Fanfei Meng, Xin Zhang, Kun Luo, Xiaoxing Su, Yan Lei, Jiuhui Xu, Jingbing Lou, Han Wang, Zhiye Du, Yunfan Wang, Yuan Li, Tingting Ren, Jie Xu, Xin Sun, Xiaodong Tang, Wei Guo

**Affiliations:** a Musculoskeletal Tumor Center, Peking University People's Hospital, No. 11 Xizhimen South Street, Xicheng District, Beijing 100044, China; b State Key Laboratory of Membrane Biology, Institute of Zoology, Chinese Academy of Sciences, No. A3 Datun Road, Chaoyang District, Beijing 100101, China; c Shanghai OrigiMed Co., Ltd, Shanghai, No. 3576 Zhaolou Road, Minhang District, Shanghai, 201112, China; d Berry Oncology Corporation, Fuzhou, 350200, China; e Pathology Department, Peking University Shougang Hospital, No. 9 Jinyuanzhuang Road, Shijingshan District, Beijing, 100144, China; f Radiology Department & Nuclear Medicine Department, Peking University People's Hospital, No. 11 Xizhimen South Street, Xicheng District, Beijing, 100044, China

**Keywords:** Osteosarcoma, Metastases, Single-nucleotide variation (SNV), Structural variation (SV)

## Abstract

**Background:**

Osteosarcoma initially metastasing to bone only shows distinct biological features compared to osteosarcoma that firstly metastasizes to the lung, which suggests us underlying different genomic pathogenetic mechanism.

**Methods:**

We analyzed whole-exome sequencing (WES) data for 38 osteosarcoma with paired samples in different relapse patterns. We also sought to redefine disease subclassifications for osteosarcoma based on genetic alterations and correlate these genetic profiles with clinical treatment courses to elucidate potential evolving cladograms.

**Results:**

We investigated WES of 12/38 patients with high-grade osteosarcoma (31.6%) with initial bone metastasis (group A) and 26/38 (68.4%) with initial pulmonary metastasis (group B), of whom 15/38 (39.5%) had paired samples of primary lesions and metastatic lesions. We found that osteosarcoma in group A mainly carries single-nucleotide variations displaying higher tumor mutation burden and neoantigen load and more tertiary lymphoid structures, while those in group B mainly exhibits structural variants. High conservation of reported genetic sequencing over time in their evolving cladograms.

**Conclusions:**

Osteosarcoma with mainly single-nucleotide variations other than structural variants might exhibit biological behavior predisposing toward bone metastases as well as better immunogenicity in tumor microenvironment.

## 1 Introduction

High-grade osteosarcoma is a bone-forming tumor characterized by the presence of an extracellular osteoid matrix produced by cancer cells [1]. Despite several decades of clinical trials, osteosarcoma is still treated with pre- and postoperative chemotherapy as well as surgical resection of the tumor as definitive local treatment [2,3]. Cases of relapse with lung or bone metastasis fare less well, with only approximately 10%–40% of patients alive at 5 years [[4], [5], [6]].

According to the series of Cooperative Osteosarcoma Study Group (COSS) trials, metastasis most commonly develops in the lungs (81.4%) and then in bones (7.8%) but rarely in lymph nodes [6,7]. There is a small subset of osteosarcoma patients who first experience relapse with distal skeletal metastasis alone, which has been assumed to be different with regard to clinical and biological features compared to those who experience first relapse involving the lung [7,8]. It is noteworthy that this population is different from those with local recurrences involving disputable suspicion of former surgical margins [9]. According to Aung et al. [10] and Bacci et al. [7], the prognosis of patients who relapse with bone metastasis alone is worse than that of patients who relapse with lung metastasis. However, those with single, resectable and late-relapsed skeletal metastasis (usually ≥2 years) have almost the same or even better outcomes as patients with lung metastasis [7,10]. Unfortunately, there is almost no basic research on exploration of the genetic occurrence and development of this group of osteosarcoma due to the rarity of the disease.

Next-generation sequencing (NGS) has provided much insight into osteosarcoma, which is characterized by significant structural variations (SVs), including copy number alteration (CNA), chromothripsis, kataegis and loss of heterozygosity (LOH), with few point mutations [11,12]. To date, relatively few studies have systematically evaluated the evolution of osteosarcoma using matched samples [13] not to mention the population with initially bone metastasis only. This gap has motivated us to explore tumor biological characteristics and predictive biomarkers for personalized therapy for this unique subset.

Therefore, in this study, we analyzed whole-exome sequencing (WES) data for 38 metastatic osteosarcoma patients, of whom 12 initially experienced relapse with skeletal metastasis (defined as group A). Eight of the 12 patients had paired samples (primary lesions and bone metastatic lesions) with which we were able to identify and compare differences between this cohort and 26 contemporary patient samples with lung metastasis for the first time (defined as group B), 7 of whom also had paired samples. We also sought to redefine disease subclassifications for osteosarcoma based on genetic alterations to guide treatment strategies, and we correlated these genetic profiles with clinical treatment courses and prognosis to elucidate potential evolving cladograms.

## 2 Methods and materials

### 2.1 Study design

This study was performed after approval from the institutional review board of Peking University People's Hospital (PKUPH). The study was in accordance with Declaration of Helsinki and International ethical guidelines of biomedical research. This trial was registered in the Medical Ethics Committee of Peking University People's Hospital on June 25th, 2019 (IRB no. 2019PHB139-01). The trial was registered in Clinicaltrials.gov with identifier no. NCT03997747. All the patients included in this study had signed consents to participate in this study in Chinese. Consents for publication had been obtained from all the patients included in this study in Chinese.

From May 1st to October 1st, 2021, continuous patients who met the following criteria were included: 1) histologically confirmed high-grade osteosarcoma; 2) operable primary and metastatic lesions which both received resection in our institute with samples available for WES. We excluded those patients without complete clinical information, those with unqualified specimens, those initially metastatic to other sites other than bone or lung, as well as those lost to follow-up. Finally, 38 osteosarcoma patients with 58 samples were included in the analysis.

For bioinformatics analysis, we tried to 1) identify genomic aberrations between patients who firstly relapsed in skeletal metastasis (group A) and patients who relapsed with lung metastasis for the first time (group B); 2) detect tumor heterogeneity between primary and their matched metastatic lesions; 3) investigate the tumor evolution models and patients’ prognosis in different groups; 4) comprehensively investigate the tumor microenvironment (TME) spatial heterogeneity in selected cases, and additionally, to determine their relevance to tertiary lymphoid structures (TLSs) using an multiplex immunohistochemistry (*m*-IHC) panel consisting of designed surface and intracellular markers (CD68, CD163, CD206, IRF8, CD3, CD8, PD-L1, CD20, CD56, B7-H3, CD47, CD4).

### 2.2 Tumor specimen processing and genomic DNA sequencing

Formalin-fixed, paraffin-embedded (FFPE) samples were used to produce 5-mm-thick slides. The pathologist evaluated the hematoxylin and eosin (HE) slides of each specimen and ensured that the tumor content was more than 20%. DNA from FFPE tumor tissues and matched blood was obtained by using QIAamp DNA FFPE Tissue Kit and QIAamp DNA Blood Midi Kit (Qiagen, Hilden, Germany), respectively. Agarose gel electrophoresis was used to examine the quality and integrity of the DNA.

Genomic DNA was prepared for paired-end library construction using a QIAamp DNA FFPE Tissue Kit and QIAamp DNA Blood Midi Kit (Qiagen, Hilden, Germany). The libraries were captured by using the NGS-based WES of OrigiMed (Shanghai, China). Targeted genomic regions were sequenced with an Illumina NovaSeq 6000.

### 2.3 Genomic alteration detection

Genomic alterations were identified as follows. Single-nucleotide variations (SNVs) were identified based on a matched normal, a panel of normals (PoN) and a common population variant resource containing allele-specific frequencies by MuTect (v1.7). And then the variants were removed by matching in the panel of population polymorphisms such as dbSNP build 155、ExAC and 1000 Genomes data with minor allele frequency (MAF) greater than 0.01. To detect breakpoints of large deletions and medium-sized insertions from paired-end short reads, PINDEL (V0.2.5) was used. The functional impact of genomic alterations was annotated by SnpEff3.0. Copy number variation (CNV) regions were identified by Control-FREEC (v9.7) with the following parameters: window = 50000 and step = 10000. Gene fusions were detected through an in-house-developed pipeline. Gene rearrangements were assessed by Integrative Genomics Viewer (IGV).

The CNV region was further analyzed by the Genomic Identification of Significant Targets in Cancer (GISTIC2.0) algorithm [14]. GISTIC2.0 assigned a G-score to each aberration, which was defined based on the amplitude and frequency of its occurrence across samples. Then, the *Q*-value of aberrant regions was calculated, and the region with a *Q*-value <0.25 was significant. $-{\mathrm{Log}}_{10}\left(Q-value\right)$ ≥0.6 and ≤–0.6 were assigned as the thresholds for amplification and deletion [15].

### 2.4 Phylogenetic tree and tumor phylogenetics analysis

All SNVs were applied to shape phylogenetic trees by Lineage Inference for Cancer Heterogeneity and Evolution method (LICHeE) [16], which makes use of the somatic SNV patterns of samples and their variant allele frequencies (VAFs) as lineage markers to build the tree. “Root mutation” was defined as a mutation located on the trunk of the phylogenetic tree for each patient.

### 2.5 Mutational signature analysis

Somatic SNVs were divided into 96 trinucleotides (mutated base plus its sequence context) by 16 possible flanking nucleotide contexts. With the 30 published COSMIC mutation signatures [17] as a reference, we used deconstrucSigs (version 1.8.0) to obtain the signature probability matrix. The result was visualized in ggplot2 (version 3.3.2).

### 2.6 Subclonal evolution analysis

According to reference counts, variant counts, copy numbers, and VAF of each somatic mutation, we used PyClone to infer putative clonal clusters and estimate their cellular prevalence [18]. The results were applied for evolution analysis of subclones with Conality Inference in Tumors Using Phylogeny method (CITUP) [19] and visualized by Timescpe (version 1.10).

### 2.7 Multiplex immunohistochemistry

To investigate the immunologic landscape, 6 full-face FFPE patient samples were randomly selected from group A (3 patients) and group B (3 patients; Table S1). H&E-stained tissue sections were respectively reviewed by two senior pathologists (SDH and SKK) to identify the regions of interest (ROIs). These represent tumor positions relative to surrounding tissue. The serially sectioned tissue was stained with the *m*-IHC panel. A total of 1800 high-power fields were imaged across all patient ROIs. A supervised image analysis system (QuPath) was used to obtain cell phenotyping data based on the pattern of marker expression.

m-IHC was achieved with a PANO 7-plex IHC kit (cat 10004100100; Panovue, Beijing, China). FFPE sections of samples were dewaxed and rehydrated. For the first and subsequent rounds, antigen retrieval was performed in EDTA (pH 9.0) buffer using a microwave (100–150 mW, 10 min). The slides were cooled to room temperature, washed with TBST/0.1% Tween (3 times, 5 min) and incubated with H_2_O_2_ (3%) for 10 min. The slides were washed and blocked with blocking buffer for 10 min. Three panels were used to perform *m*-IHC. Nuclei were stained with 4′,6-diamidino-2-phenylindole (DAPI), and slides were mounted with medium.

### 2.8 Statistical analysis

Statistical analyses were performed using R software. The median and quartiles were used to describe demographic data and clinical outcomes. T test was used to evaluate differences in normally distributed data, while Mann–Whitney U (Wilcoxon) test was used to assess differences in non-normally distributed data. The chi-square test was employed to evaluate differences in enumeration data. Overall survival and progression-free survival were estimated by Kaplan–Meier analysis. Differences were significant at *P* < 0.05.

## 3 Results

Our cohort consisted of 12/38 patients with high-grade osteosarcoma (31.6%) with initial bone metastasis (group A) and 26/38 (68.4%) with initial pulmonary metastasis (group B), of whom 15/38 (39.5%) had paired samples of primary lesions and metastatic lesions (Table 1, Fig. 1A–C, and Table S1). For our discovery set, WES was at a median depth of ∼591 × (range, 268–1239) using paired constitutional DNA from peripheral blood. Five of 15 (33.3%) paired tumor samples were derived from pretherapeutic biopsies. In other words, these samples presented the initial characteristics of their tumor status. We first assessed genomic complexity within this cohort along with clinical progression in these cases. These data were then partly integrated with transcriptome and immunostaining results to characterize the genomic landscape of our osteosarcoma cohort.

**Fig. 1:**
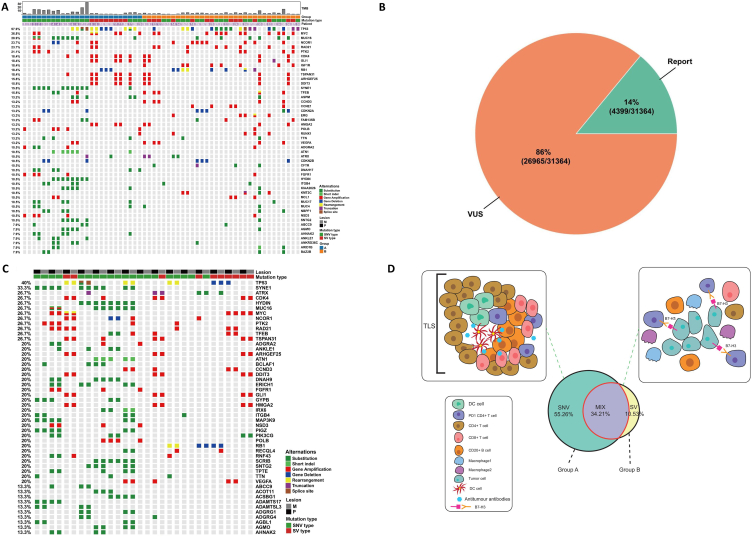
The result of whole exome sequencing (WES) in 38 metastatic osteosarcoma (OS) patients. **A** The clinical, pathologic, and predominant genomic landscape characteristics of all OSs with DNA sequencing. **B** The report mutation and VUS mutation of 38 patients with osteosarcoma. Only 14% (4399/31365) of the genomic alterations were reported mutations while 86% (26966/31365) were not reported in any literatures. **C** Mutation landscape of paired primary and metastatic osteosarcoma. **D** The diagram showed the tumor immune microenvironment of osteosarcoma metastasis. The karyotype of tumor cell included SNV type, structural variant (SV) type, and mixed type. A large portion of the group A was of single-nucleotide variation (SNV) type, while group B were mainly SV type and mixed type. TLSs were found at the edges and margins of the tumor in group A, while strongly positive expression of B7-H3 was found in group B. Most of the genes participated in the DDR pathway and Notch pathway in group A.

### 3.1 Clinicopathologic characteristics and genomic landscape

The clinical demographics of the 38 high-grade osteosarcoma patients are summarized in Table 1. The clinical, pathologic, and predominant genomic landscape characteristics of all osteosarcoma with DNA sequencing are shown in Fig. 1A. Therefore, subsequent WES data analyses were performed on 58 osteosarcoma samples from 38 patients. With a median follow-up time of 26.0 months (Q1, Q3, 16.8, 39.0), we did not find any obvious significant difference in progression-free survival (*P* = 0.33) or overall survival (*P* = 0.06) between these two groups (Figure S5).

We defined driver mutations in established cancer genes, which mainly include SNVs and SVs, to describe the genomic landscape for the whole population. It is interesting that our cohorts seemed to show diverse biological behaviors based on the main genomic manifestations. Our population contained more point mutations other than historical high-risk osteosarcoma genetic sequencings published [13,20]. Consequently, we adopted a novel strategy to re-subclassify our tumors, restricting our focus to high-confidence variants (see Methods and Materials section). A large portion of the group A were of the SNV type. Group B showed similar manifestation as reported for high-risk osteosarcoma, involving genomic amplifications as well as fusions or mixed type (which we called the SV type), with few recurrent point mutations (Fig. 1D).

Somatic alterations detected by our methods in the 38 metastatic patients are shown in Fig. 1A. Among genes commonly mutated, TP53 and MYC were identified in 22/58 and 14/58 patients respectively (57.9% and 36.8%; Fig. 1A and Table S2). However, for this cohort of patients with osteosarcoma (OS), we did not specifically design our panel to identify TP53 intron 1 rearrangements, as has recently been reported in OS [12], and more TP53 mutation might be missing due to the WES approach. Nevertheless, we noticed that for WES, only 14% (4399/31365) of the genomic alterations are reported mutations, whereas 86% (26966/31365) are not reported in the literature (Fig. 1B); it is unclear whether these alterations influence the occurrence and evolution of OS.

The burden of coding indels and substitutions across the 58 tumor samples varied from 2 to 1482 mutations per tumor (median 84). The highest coding mutation burden (32.9 mut/Mb) was observed in patient WJY, who initially presented with bone metastasis with a time interval from diagnosis to metastasis longer than 24 months and whose tumor exhibited MSI.

### 3.2 Diverse genomic characterization between osteosarcoma initially metastatic to bones and to lungs

We showed the mutation profiles between group A and group B in Fig. 2A. Surprisingly, group A mainly exhibited gene substitutions and short indels (green mark); group B mainly presented with gene amplifications, deletions and rearrangements (red mark). To describe this phenomenon more precisely, we reclassified our population into two genomic types: SNVs, accounting for more than 40% of all genomic alterations were gene substitutions and short indels; SVs, accounting for more than 40% of all genomic alterations were amplifications, homozygous deletions and breakpoints that disrupted genes or generated gene fusions. For those with both SNVs and SVs of more than 40%, we classified them as the mixed type. In fact, a large portion of our population had the mixed type (17/38, 44.7%, 1/12 8.3% for group A & 16/26 61.5% for group B; Fig. 1C). Thus, to highlight patients with distinct genomic manifestations from group A, we further integrated the mixed type into the SV type. We compared all these patients’ genomic changes (see Table 2). Based on chi-square analysis, we observed obverse statistical disequilibrium for the differences between two groups (*P* = 0.018), which suggests that the biological behavior from the perspective of genomic manifestations was distinct for these two groups of population. It should be noted that we further compared the TMB, neoantigen, MSI and human evolutionary divergence (HED) status between the two groups. Although it was a small sample sizes, we observed diverse differences in TMB (*P* = 0.01) and neoantigen status (*P* = 0.0028) respectively (Fig. 2C and D).

**Fig. 2:**
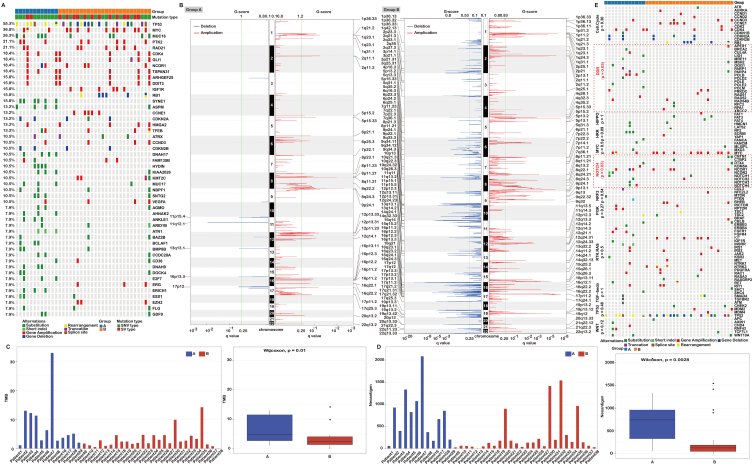
The differences between group A and group B. **A** Mutation profiles of group A and group B. **B** Copy-number profile of group A and group B. Copy number variation (CNV) amplifications and deletions were more frequent in group B. **C** The barplot and boxplot of TMB of group A and group B. The median tumor mutation burden (TMB) was 4.85 (Q1, Q3, 2.75, 11.98) for group A while for group B, the median TMB was only 2.4 (Q1, Q3, 1.38, 4.45). **D** The barplot and boxplot of neoantigens of group A and group B. the median amounts of neoantigen in group A was almost six times more than group B with 743 (316.5, 1034.5) versus 128.5 (49, 200.5). **E** The profiling pathway of group A and group B. Diverse difference with most of the genes focused on DDR pathway (*P* = 0.03), Notch pathway (*P* = 0.03), while no diverse difference were found in cell-cycle, TP53, IGF-beta, MYCHRR, NOTCH and so on.

**Table 1:** Clinical demographics of the 38 metastatic osteosarcomas.

Characteristic	Group A	Group B	Statistical method	*P* value
*n* (%)	12 (31.6)	26 (68.4)		
Age at diagnosis (in years)			T test	0.060
Range	6–61	6–57		
Median (Q1, Q3)	19.5 (13.25, 38.75)	14 (11, 17.5)		
Gender, *n* (%)			Chi-square	1.000
Male	9 (23.7)	20 (52.6)		
Female	3 (7.9)	6 (15.8)		
Paired primary osteosarcomas, *n* (%)			Chi-square	0.033
Yes	8 (21.1)	7 (18.4)		
No	4 (10.5)	19 (50.0)		
Primary sample site, *n* (%)			Chi-square	0.280
Femur	8 (21.1)	16 (42.1)		
Humerus	0 (0.0)	3 (7.9)		
Tibia	2 (5.3)	6 (15.8)		
Pelvis	1 (2.6)	1 (2.6)		
Spine	1 (2.6)	0 (0.0)		
Metastatic sample site, *n* (%)			Chi-square	0.000
Femur	2 (5.3)	3 (7.9)		
Fibula	2 (5.3)	1 (2.6)		
Tibia	0 (0.0)	2 (5.3)		
Pelvis	4 (10.5)	0 (0.0)		
Rib	1 (2.6)	0 (0.0)		
Sacrum	1 (2.6)	0 (0.0)		
Spine	2 (5.3)	1 (2.6)		
Lung	0 (0.0)	19 (50.0)		
Adjuvant therapy, *n* (%)				
First-line treatment	11 (29.0)	25 (65.8)	Chi-square	0.538
Second-line treatment	9 (23.7)	22 (57.9)	Chi-square	0.656
Third-line treatment	5 (13.2)	10 (26.3)	Chi-square	1.000
Fourth-line treatment	2 (5.3)	5 (13.2)	Chi-square	1.000
Vital status, *n* (%)			Chi-square	0.108
Alive	7 (18.4)	22 (57.9)		
Dead	5 (13.2)	4 (10.5)		
PFS (month)			T test	0.596
Range	0–48.23	2.23–34.43		
Median (Q1, Q3)	12.4 (7.45, 21.22)	13.95 (8.00, 19.75)		
Overall survival or follow-up time (month)			T test	0.856
Range	7–59	9–54		
Median (Q1, Q3)	23.5 (15.25, 46.5)	27 (16.75, 38.25)		

HED score >7.887, defined as HED high; HED score ≤7.887, defined as HED low. HED, human leukocyte antigen-I evolutionary divergence; PFS, progression free survival.

**Table 2:** The difference of osteosarcoma patients’ genomic alterations between group A and group B.

	Group A	Group B	Statistical method	*P* value
*n* (%)	12 (31.6)	26 (68.4)		
Genomic type, *n* (%)			Chi-square	0.0180
SNV	10 (26.3)	11 (28.9)		
SV or mixed	2 (5.3)	15 (39.5)		
TMB			Wilcoxon	0.0100
Range	1.1–32.9	0–14.2		
Median (Q1, Q3)	4.85 (2.75, 11.98)	2.4 (1.38, 4.45)		
Neoantigen			Wilcoxon	0.0028
Range	57–2084	0–1539		
Median (Q1, Q3)	743 (316.5, 1034.5)	128.5 (49, 200.5)		
MSI, *n* (%)			Chi-square	0.3160
MSS	11 (29.0)	26 (68.4)		
MSI-H	1 (2.6)	0 (0.0)		
HED-status, *n* (%)			Chi-square	1.0000
Low	10 (26.3)	22 (57.9)		
High	2 (5.3)	4 (10.5)		

HED score >7.887, defined as HED high; HED score ≤7.887, defined as HED low. SNV, single-nucleotide variation; SV, structural variation; TMB, tumor mutation burden; MSI, microsatellite instability; MSS, microsatellite stability; MSI-H, high microsatellite instability; HED, human leukocyte antigen-I evolutionary divergence.

The copy number variation region was further analyzed, and we found that CNV amplifications and deletions were more frequent in group B than in group A (Fig. 2B). We had incorporated CNV into different chromosomes by different relapse patterns (shown in Fig. 2B) and noticed obvious different manifestation.

The mutation burden of osteosarcoma was similar reported as pancancer analysis [21]. However, the median TMB was 4.85 (Q1, Q3, 2.75, 11.98) and 2.4 (Q1, Q3, 1.38, 4.45) for group A and group B, respectively (Fig. 2C). The median amount of neoantigen in group A was almost six times more than that in group B, at 743 (316.5, 1034.5) versus 128.5 (49, 200.5; *P* = 0.0028; Fig. 2D). We compared all mutations in cell signaling pathway between the two groups and found diverse distinct gene pathways participating in the DNA damage response (DDR) (*P* = 0.03), and Notch (*P* = 0.03), whereas no difference in the cell cycle, TP53, IGF-beta, MYC, HIPPO, RTK or RAS pathways (Fig. 2E).

We analyzed the gene signature profiles of all 38 patients based on the literatures for renal cell carcinoma or non-small cell lung cancer. However, mutation spectra revealed significant differences for signature 1 between the groups, mainly referring to age (Fig. S2), which was in accordance with recent publications on genomic differences between pediatric and adult sarcomas by targeted panel sequencing of 7494 sarcomas representing 44 histologies [22,23]. However, we further compared the age distribution between these groups of patients with the Mann–Whitney *U* test (Fig. S3) but did not observe any significant difference, with a *P* value of 0.06 (detailed information could be observed in Table S1).

### 3.3 High conservation of reported genetic sequencing over time in most osteosarcomas

For the 15 patients with paired primary and metastatic samples, we performed tumor phylogenetics to identify the basis of metastasis (Fig. 3). Of the 15 patients, 8/15 (53.3%) were from group A and 7/15 (46.7%) from group B; their genomic manifestations of non-silent mutations are depicted in Fig. 1C. Other than prominent mutation landscape diversity between group A and group B (Fig. 3A), we did not observe any reported alterations' differences between primary and metastatic specimens for genomic landscapes (Fig. 1C), neither for TMB (Fig. S2A), neoantigens (Fig. S2B) nor profiling pathways (Fig. S2C). However, it was difficult to precisely define whether these cases involved linear or parallel progression models (Fig. S4) [24]. Most of our cases, regardless of group A or group B, were more similar to macroevolution as literature’ description [25]. In the present study, we employed two methods to describe these phylogenetics to identify the origin of metastasis (Fig. 3 and Fig. S4), which exhibited the subclonal evolution model (mutation- and copy number-based) and multi-sample lineage trees based on the VAF of somatic SNVs respectively. We utilized the root/branch ratio (dN/dS ratio) for calculation [26] and found that groups A and B were not statistical different (*P* = 0.46).

**Fig. 3:**
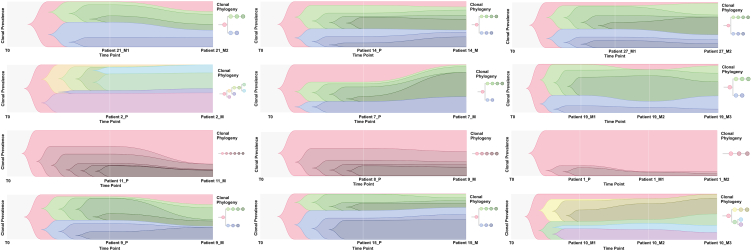
The evolutionary process of subclones of the 12 osteosarcoma with paired samples.

### 3.4 A distinctive immune ecosystem in osteosarcoma with initially bone metastasis based on multiplex immunohistochemistry

TLSs are an important biomarker for identifying selected soft tissue sarcomas that might show a response to immune checkpoint inhibitors (ICIs). Surprisingly, according to *m*-IHC of 3 randomly selected patient samples from group A, one (HXD) showed TLSs at the edges and margins of the tumor (shown in Fig. 4A); 3 from group B lacked TLSs or CD3^+^CD8^+^ T cells (Fig. 4B). It is also interesting that all patients in the vacant district of PD-L1 (programmed cell death ligand 1) expression usually showed high intensity and strongly positive expression of B7-H3 (Fig. 4A, B & 4C; *P* = 0.0043). However, the patient (HXD) with multiple TLS districts of an immunogenic state exhibited poor staining of B7-H3 (Fig. 4A), the mechanism of which should be further investigated. Findings between gene expression profilings and immunohistochemistry analysis were complementary and showed modest correlation, which suggested that group A patients seemed to be in a more immunogenic state than group B.

**Fig. 4:**
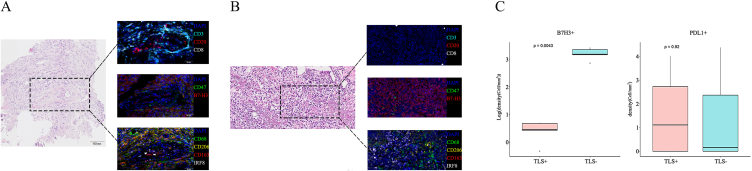
The multiplex immunohistochemistry results of osteosarcoma patients.

## 4 Discussion

The first site of metastasis in osteosarcoma patients is usually the lung. Skeletal metastasis has rarely been described as having distinct biological and clinical behaviors and with diverse outcomes [7,10,27]. We conducted a comprehensive genomic and immune characterization of primary and paired-metastatic osteosarcoma specimens to determine the molecular bases for osteosarcoma first metastasizing to bone alone. Furthermore, to compare differences, we conducted WES for 26 contemporary patient samples firstly metastasizing to the lung in the same institution with paired samples. To our knowledge, this might be the largest sample with paired specimens to demonstrate the genomic landscape and potential evolving cladograms over time for this special group (referred to as group A in our study). We observed diverse differences in genomic manifestations for these two groups of population. First and most importantly, enrichment of SNVs in bone metastatic patients and focal clustered SVs obviously occurred in those with pulmonary metastasis, suggesting that oncogenesis may be more driven by catastrophic chromothripsis events in osteosarcoma with lung metastasis. Second, our set of 15 primary and metastatic matched osteosarcoma samples showed high conservation of reported genetic sequencing profilings; thus, although osteosarcoma shows significant genomic complexity with SVs and genomic instability, many of the genomic events are early events that remain stable over time. Third, osteosarcoma firstly metastasizing to bone might initially benefit from immunotherapy because it tends to have more non-reported point mutations and its TME manifests as a more inflamed immune state.

Osteosarcoma is characterized by complex genome instability and a high level of genetic heterogeneity [11]. Osteosarcoma is also characterized by specific alterations in tumor suppressors and oncogenes, including p53, Rb, CDK4, MDM2, and MYC [28,29]. The majority of the resulting genetic alterations are associated with copy number changes and genome rearrangement [11,12,30,31]. Whether the observed SVs are truly targetable vulnerabilities remains to be fully explored [12], and clinically, it is almost certain that combination approaches need to be developed, as single agents are unlikely to lead to significant tumor regression. For many decades, treatment for osteosarcoma has strongly focused on the development of cytotoxic and cytostatic drugs, frequently leading to disappointing results due to inter- or intratumoral heterogeneity [32]. The development of immunotherapy has revolutionized the status quo for inducing durable responses and improving osteosarcoma [32]. However, in recent trials of ICIs, only a limited number of osteosarcoma patients have derived meaningful clinical benefits, without a statistical advantage for the whole population [[33], [34], [35], [36], [37]]. Selecting patients who may benefit from immunotherapies has become an urgent question that needs to be addressed. According to our study, those osteosarcoma with initial bone metastasis alone might benefit more from immunotherapy.

It is worth noting that between our two groups of patients, we noticed that gene signature 1 had distinct expression, with a *P* value of 0.004, which indicated that the age distribution might confound the outcome. We further listed all these patients’ ages and compared their distribution between the two groups in an independent-sample t-test and did not find a significant difference (*P* = 0.06) (Fig. S3). Several other studies [11,38,39] have revealed distinct molecular characteristics for pediatric and adult OSs with small sample sizes. Recently Gounder et al. [23] had verified significant differences in genomic alterations using an age cutoff of ≤30 years in osteosarcoma by larger sample size (*N* = 459). Copy number variations differed between pediatric patients and adults with sarcomas, which indicated us that this diverse observations from genomic landscapes between these two groups might partly be influenced by age. However whether relapse patterns or age eventually contributing to this phenomenon needs to be further investigated in basic experiment.

Whole genome doubling (WGD) events across different sarcomas was supposed to be associated with decreased overall survival [23]. In the present study, the phylogenetic relationship between the regional biopsies of a given sample was inferred from mutation data using LICHeE [40], PyClone [17] and CITUP [18]. We employed two methods to describe these phylogenetics to identify the origin of metastasis (Fig. 3 and Fig. S4), which exhibited more like macroevolution [25]. Tumor macroevolution was also found to be driven by chromothripsis, whereby a single catastrophic mutational event is thought to be responsible for the generation of highly complex genomic rearrangements involving dozens of breakpoints. We had tried to differ different phylogenetic processes between group A and group B and did not find any obvious difference.

Several limitations in the current study should be acknowledged. First, most specimens were collected retrospectively from formalin-fixed paraffin-embedded slices; thus, our methods involved WES for only selected cases including fresh specimens directly after surgical resection, as detected by RNA-seq. Wu et al. [20] found that unexpressed mutations tend to occur in genes that have low expression or with low VAFs. Thus, our WES only analysis might miss abundant genetic information for these patients. Second the sample size was relatively small. Although we sought to validate our assumption by larger sample sizes, the clinical information was not as meticulous and complete in The Cancer Genome Atlas (TCGA) and the Therapeutically Applicable Research to Generate Effective Treatments (TARGET) database with only scarce cases without statistical comparison. Besides, there was technical limitation due to the difficulty in processing bone tissue resulting in not all samples sequencing successful. Finally, genomic subclassifications were identified in this study, implying that immunotherapy might be effective for osteosarcoma initial developing bone metastasis, though we have not yet verified it in more patients in the form of clinical trials, which further should be intelligently designed.

## 5 Conclusions

Osteosarcoma initially metastasizing to bone mainly showed more SNVs, whereas those first metastasizing to the lung mainly had SVs. This study suggests that osteosarcoma patients with initial genetic landscapes of mainly SNVs should be paid more attention on monitoring bone metastases; immunotherapy might be a promising therapeutic option for this group of patients.

## Ethic statement

This study was performed after approval from the institutional review board of Peking University People's Hospital (PKUPH). The study was in accordance with Declaration of Helsinki and International ethical guidelines of biomedical research. This trial was registered in the Medical Ethics Committee of Peking University People's Hospital on June 25th, 2019 (IRB no. 2019PHB139-01). The trial was registered in Clinicaltrials.gov with identifier no. NCT03997747. All the patients included in this study had signed consents to participate in this study in Chinese. Consents for publication had been obtained from all the patients included in this study in Chinese.

## Author contribution statement

Lu Xie, Zhenyu Cai, Hezhe Lu, Fanfei Meng, Xin Zhang, Kun Luo, Xiaoxing Su, Yan Lei, Jiuhui Xu, Jingbing Lou, Han Wang, Zhiye Du, Yunfan Wang, Yuan Li, Tingting Ren, Jie Xu, Xin Sun, Xiaodong TangORCID, Wei Guo: Conceived and designed the experiments; Performed the experiments; Analyzed and interpreted the data; Contributed reagents, materials, analysis tools or data; Wrote the paper.

## Data availability statement

Data associated with this study has been deposited at “National Genomics Data Center of China” under the accession number CNP0001734.

## Figures and Tables

**figs1:**
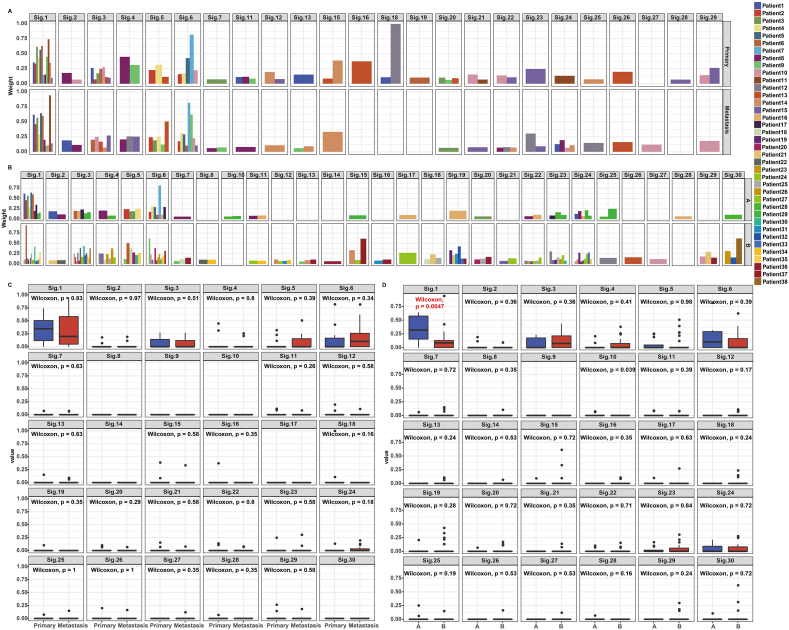


**figs2:**
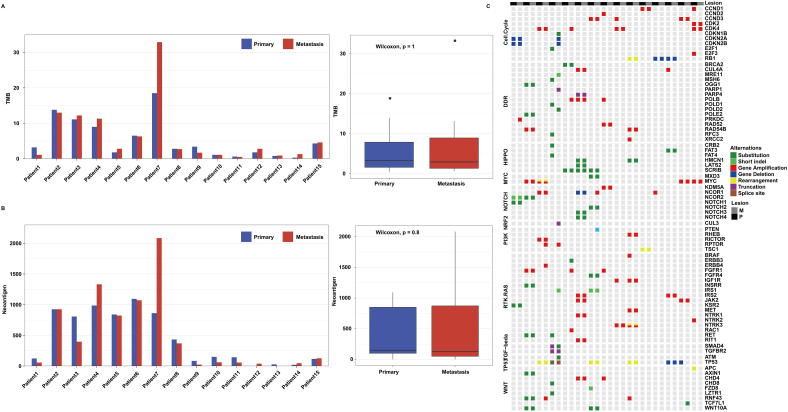


**figs3:**
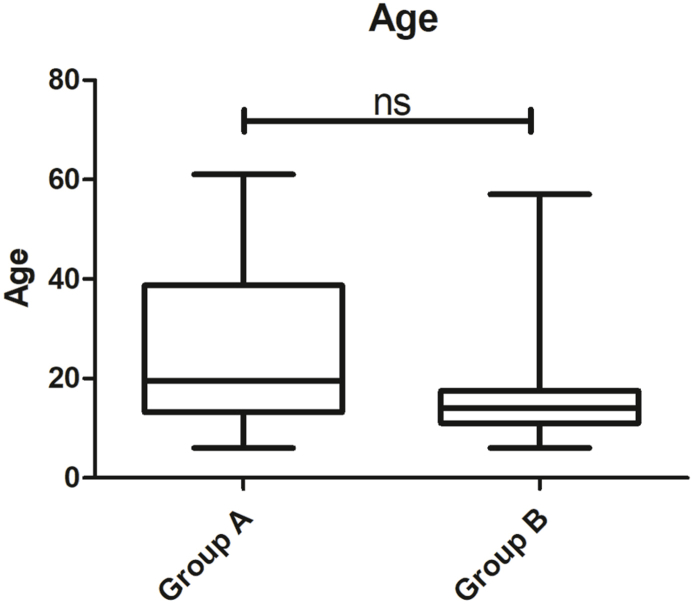


**figs4:**
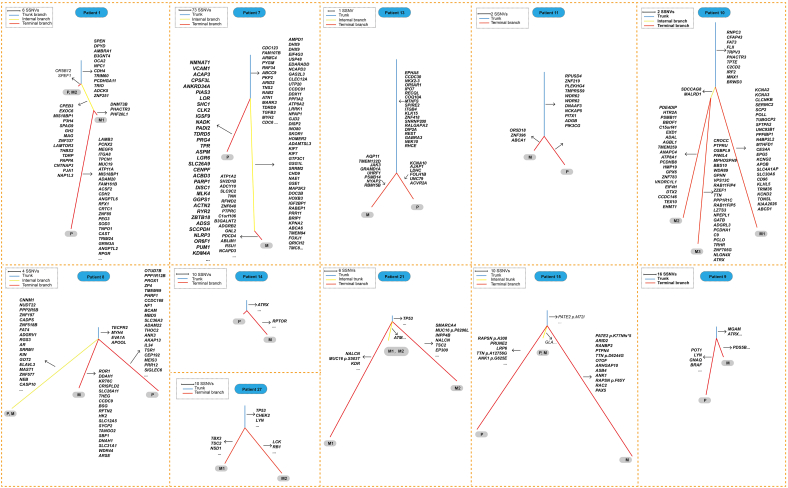


**figs5:**
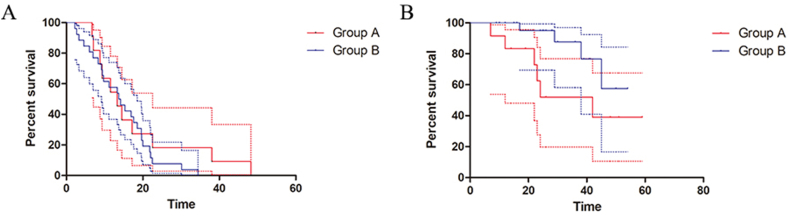


## Abbreviations

CITUP, Conality Inference in Tumors Using Phylogeny method; CNA, copy number alteration; COSS, Cooperative Osteosarcoma Study Group; DAPI, 4′,6-diamidino-2-phenylindole; DDR, DNA damage response; dMMR, DNA mismatch repair deficiency; FFPE, formalin-fixed, paraffin-embedded; HE, hematoxylin and eosin; HED, human evolutionary divergence; ICI, immune checkpoint inhibitor; IGV, Integrative Genomics Viewer; LICHeE, Lineage Inference for Cancer Heterogeneity and Evolution method; LOH, loss of heterozygosity; *m*-IHC, multiplex immunohistochemistry; MSI, microsatellite instability; NGS, next-generation sequencing; MSS, microsatellite stability; OS, osteosarcoma; PERCIST, PET Response Criteria in Solid Tumors; PET/CT, positron emission tomography/computed tomography; PFS, progression free survival; PKUPH, Peking University People's Hospital; PMR, partial metabolic response; RNA-seq, RNA sequencing; ROI, region of interest; SNV, single-nucleotide variation; SV, structural variation; TARGET, Therapeutically Applicable Research to Generate Effective Treatments; TCGA, The Cancer Genome Atlas; TLS, tertiary lymphoid structure; TMB, tumor mutation burden; TME, tumor microenvironment; VAF, variant allele frequencies; WES, whole-exome sequencing; WGD, whole genome doubling.

## Appendix B Supplementary data

Table S1 Detailed clinical demographics Table S1 Detailed clinical demographics
